# The Impact of Herbs and Spices on Increasing the Appreciation and Intake of Low-Salt Legume-Based Meals

**DOI:** 10.3390/nu11122901

**Published:** 2019-12-01

**Authors:** Anestis Dougkas, Marine Vannereux, Agnès Giboreau

**Affiliations:** Institut Paul Bocuse Research Centre, BP 25-69130 Ecully CEDEX, France; marinevannereux@gmail.com (M.V.); agnes.giboreau@institutpaulbocuse.com (A.G.)

**Keywords:** liking, salt, herbs and spices, legumes, appetite, eating behavior, living lab

## Abstract

Flavoring using blends of herbs and spices (H&S) instead of salt may be a promising approach to increase legume consumption and reduce salt content in foods. This study examines the effects of H&S on the appreciation and intake of low-salt legume-based dishes in a real ecological eating environment. Four mezzes with standard (0.8% *w*/*w*) (S) or lower (0.4% *w*/*w*) (LS) salt level and with or without H&S (LSHS, SHS) were developed. In a randomized cross-over trial, 94 participants attended four sessions, one week apart and received the four variants as a starter during lunch. Overall liking, food intake, and appetite ratings (visual analogue scale, VAS) were assessed during lunch. A follow-up study (*n* = 130) was performed where the four variants were evaluated, and overall liking was measured at the same session. Overall liking and taste scores of SHS were significantly higher compared with LS (*p* = 0.04 and *p* = 0.03, respectively), but there were no significant differences between LSHS and S. However, there were no differences in energy intake or in appetite ratings among the products. Overall appreciation was similar between the low-salt with H&S and the standard-salt mezze, suggesting that the addition of H&S is a feasible strategy for achieving a 50% reduction in salt content without compromising hedonic appreciation.

## 1. Introduction

Along with rapid global population growth comes the challenge of ensuring a food supply of optimum quantity and quality with respect to the environment and health. A dietary pattern that is higher in plant-based foods, including vegetables and legumes, and lower in salt is related to better health and lesser environmental impact [1]. The growing presence of eating out of home in the Western diet [2,3] could reflect changes in both the demand of consumers and the approach of food providers, thereby affecting the nutritional quality of food and, consequently, consumer health. Given that commercially processed food products and restaurant foods contribute substantially to meat [4] and dietary salt intake [5,6], developing healthy meals, which are plant-based and low in salt content, is a major challenge for the food industry and restaurant sector due to its impact on foods’ sensory profile and consumer acceptance [7].

Dietary salt is an important component that is commonly used to make many food products and meals more flavorful. Nevertheless, salt intake around the world far exceeds nutritional requirements [8], which is particularly worrisome given the strong evidence that high salt intake is related to hypertension, thereby increasing the risk of cardiovascular diseases [9]. Global salt reduction programs are in place through awareness campaigns and governmental interventions [10] in line with the World Health Organization (WHO) recommendation to reduce salt intake by 30% by 2025 [11].

Furthermore, legumes are low in fat and densely packed with proteins, complex carbohydrates, fiber, and B-vitamins, and they are rich in several micronutrients, such as folate and iron [12,13]. They are also known for their association with prevention of type 2 diabetes, cardiovascular disease, hypertension, obesity, and cancer [14,15,16]. Despite the well-documented health benefits of legume consumption, the actual intake of legumes remains low [17,18]. Among the reasons for the low consumption is flavor perception, which influences food choice [19] and food intake [20]. Flavor is described as the combination of taste (gustation), smell (olfaction), and chemical burn (trigeminal sensation) [21]. Using a blend of herbs and spices to enhance the flavor and palatability of foods is a potential approach to assist consumers with reduced salt consumption, as well as to improve legume acceptance. 

Ghawi et al. [22] directly evaluated the impact of herbs and spices on enhancing liking of low-salt tomato soup. Participants were asked to rate the overall liking of three tomato soup samples: regular salt, low salt, and low salt with added herbs and spices. Overall liking, flavor, and texture liking were significantly increased only for the low salt with added herbs and spices sample after repeated exposure to the three soup variants. The addition of herbs and spices to modified versions of traditional foods could, thus, increase palatability while compensating for reductions in salt [22,23], fat, and energy [24,25]. Prior work also showed that herbs and spices could be an effective strategy to improve the flavor and increase liking and preference for foods with lower palatability such as vegetables [26,27,28,29]. Such a strategy is promising but research is still limited. In particular, the effectiveness of herbs and spices to improve overall liking and consumption of legumes remains to be tested. 

In addition, there is some evidence that herbs and spices might also play a role in appetite sensations through various potential mechanisms [30,31,32]. For instance, the perceived heat from chili peppers may be linked with increased post-meal satiety (sense of fullness) [33]. Certain components found in legumes such as protein and fiber might also influence appetite regulation. Kristensen et al. [34] showed that protein-rich meals based on beans and peas increased satiety more than protein-rich veal and pork-based meals. Meat is among the most popular choices in eating out of home [4] and, with more and more people choosing to eat away from home [35], the more traditional “full-service” restaurant sector contributes significantly to the out-of-home dining market [36]. In this setting, using herbs and spices to enhance liking of legumes while compensating for reduction of salt in a real context ecological restaurant is an area of research that needs further attention.

The aim of this study was to determine whether adding herbs and spices to low-salt legume-based dishes can increase overall liking and preferences of legumes among young adults at a single meal occasion. In addition, the study examined whether the sensory modifications using herbs and spices could influence satiation and food consumption in a real context ecological environment. The hypothesis was that the addition of herbs and spices can enhance the overall liking of low-salt legume-based dishes and increase their intake relative to plain legume-based dishes. 

## 2. Material and Methods

The study design to test the impact of using herbs and spices to increase the appreciation and intake of legume-based dishes included the following three phases:

- Phase I: recipe development and determination of the most liked herb and spice modification for use in further study and confirmation of differences in perception of saltiness and spiciness.
- Phase II: evaluation of the overall liking of the different versions of the selected recipe in different meal sessions (one week apart), as a measure of absolute liking.
- Phase III: follow-up assessment study, where all four versions of the legume-based dish were evaluated during the same session, as a measure of relative liking.

### 2.1. Phase I

#### Recipe Development 

Professional research chefs from the Institut Paul Bocuse and McCormick Flavor Company (Avignon, France) developed legume recipes with the goal to be sustainable, reproducible in terms of cooking process and seasoning, and easily standardized in terms of flavor, texture, and temperature (hot/cold). The goal is to use them for future interventions in school or university canteens. Legumes are usually consumed as an entire dish (e.g., lentil soup), side dish (e.g., salads), or snacks (e.g., spiced chickpeas), or as an ingredient in another dish (e.g., stews, casseroles, sauces) [37]. In addition to the type of meal, several other factors were considered such as the type (e.g., lentils, chickpeas, or beans) and form of legumes (e.g., whole, mashed, spread). In order to maintain standardization in taste, volume, and texture and cooking practicalities during the study visits, a mezze (small savory dish that can be served as an appetizer) with mashed legumes, a hummus-type spread, was developed with a blend of 70% chickpeas (Sabarot, France) and 30% red lentils (Sabarot, France) as the base. Chickpeas and lentils were selected after experimenting with different types of legumes with regard to texture and appearance of the final products. In addition, the blend of those legumes is a novel approach to incorporating and increasing the consumption of different types of legumes, producing diverse nutritional characteristics in the final products. For instance, lentils have a better nutrition profile in terms of fat, fiber, and protein content than chickpeas [38]; thus, it was a way to reduce the fat content in the final product. 

The base of legume was prepared by soaking 120 g of raw chickpeas in three times its quantity of water during 24 h at 4 °C in order to have 240 g of hydrated chickpeas. The chickpeas were cooked for 90 min in three times the quantity of simmering water. The red lentils were rinsed and cooked in simmering water for 12 min. No salt was used during the cooking process. After simmering, all of the ingredients were mixed thoroughly in a blender (Panasonic Ltd. MX-ZX1800SXE, France) at the highest speed for 5 min in order to obtain a smooth and homogeneous base (Table 1).

This base was produced as a single batch, vacuum-packed in different bags, and then frozen at −25 °C in order to keep high standardization of texture and product characteristics for all the sessions. The bags were thawed overnight before each session and salt, herbs, and spices were added and mixed thoroughly using a blender. The reference salt level was set at 0.8% to represent the average salt level of similar products, according to a market benchmark, and the low-salt level was set at 0.4% *w*/*w* salt content, representing a reduction of 50%. The formulation of the low-salt legume base (100 g) was modified by the inclusion of four different blends of herbs and spices as follows:

- Curcumin blend modification: curcumin (0.8 g), ginger (0.4 g), shallot (0.4 g), and garlic (0.4 g);
- Ginger modification: ginger (1 g);
- Paprika blend modification: paprika (0.6 g), tomato (0.6 g), coriander (0.4 g), and garlic (0.4 g);
- Cumin blend modification: cumin (0.4 g), shallots (0.6 g), garlic (0.4 g), and spinach coulis (10 g).

(A) Selection of the most liked herb and spice blend for the legume-based mezze 

Those four legume-based mezzes with different blends of herbs and spices were tested in a consumer test to determine the most liked herb and spice modification blend. In total, 130 consumers aged 18–35 without any relevant food allergies and intolerances, dislike of legumes, or health problems affecting taste or smell were recruited using an online invitation (database with volunteers that were previously engaged in meal studies and showed interest in participating in similar studies in the future). The test was carried out at an experimental platform, the Living Lab restaurant (real but controlled environment) of the Research Center of Institut Paul Bocuse in Écully, France. These types of restaurants provide a controlled environment that is ecologically valid from a consumer point of view and particularly relevant to study consumers’ choice and preference in real-life situations in order to consider the variability of real meal responses [39]. Written informed consent was obtained from participants before their participation in the study. Participants were assigned randomly to shared tables, where they could converse with other participants, but they were instructed not to talk to others about the test meal items. The experimental restaurant accommodated up to 25 subjects that could be served each day. Each participant was served with 30 g of each mezze as a starter dish at lunch (served 12:15 p.m.). The mezzes were served individually in identical plastic cups, numbered with a three-digit random code, and presented on the same line in a balanced order (permuted blocks of four). Participants were asked to taste the four mezzes without the need to finish any of them and to rinse their palate between samples with water and buckwheat salt-free crackers (Le Pain des Fleurs, France) in order to cleanse their palate. Overall hedonic liking was scored on a nine-point Likert scale, followed by two open-ended questions asking consumers to list positive characteristics on one side and negative characteristics on the other side. A hedonic ranking question was finally administered to collect explicit participants’ preference. 

(B) Directional paired comparisons for salt and spice intensity

Based on the initial consumer test, four versions of the most liked herb and spice modification blend for the legume-based mezze were created with different levels of salt and herbs and spices according to a 2 × 2 factorial test (low (0.4% *w*/*w*) vs. standard salt (0.8% *w*/*w*) and with or without herbs and spices) in order to assess perceptions of consumers. The four test products were as follows:

- The standard-salt legume-based mezze (**S**);
- The low-salt legume-based mezze without herbs and spices (**LS**);
- The standard-salt legume-based mezze with herbs and spices (**SHS**);
- The low-salt legume-based mezze with herbs and spices (**LSHS**).

The two-alternative forced choice test (2-AFC), a method in which two stimuli are presented, and assessors are given a criterion by which they are required to select one stimulus, was used to assess perception of saltiness and spiciness. This study was carried out at the Living Lab restaurant of Institut Paul Bocuse, as described earlier. In total, 113 new participants were recruited in this test using a similar online invitation as for phase I.A, and they were between 18 and 35 years old without any relevant food allergies and intolerances, aversion to legumes, or health problems affecting taste or smell. Written informed consent was obtained from all participants before their participation in this study. Each participant was served with 30 g of each variant. The mezzes were served individually in identical cups, numbered with a three-digit random code and presented in a balanced order (permuted blocks of two). Consumers were randomly divided into two groups to taste different pairs. The first group (*n* = 61) tested the LS/S and LSHS/SHS pairs and specified for each pair which sample was saltier. The second group (*n* = 54) tested the S/SHS and LS/LSHS pairs and specified for each pair which sample was spicier. They were asked to rinse their palate between the samples with water and salt-free crackers.

### 2.2. Phase II—Study Intervention

Appreciation of the different versions of the selected herb and spice modification.

#### 2.2.1. Subjects

New participants were recruited through an online invitation, as for the phase I tests, with the supplemental use of a flyer distributed in different universities next to the Institut Paul Bocuse and shared via LinkedIn. Participants were recruited following the inclusion and exclusion criteria described in Table 2. 

All the study phases were conducted according to the guidelines laid down in the Declaration of Helsinki and General Data Protection Regulation (GDPR), and they were approved by the Research Protocol Committee of Institut Paul Bocuse according to the ethics guidelines (Loi Jardé, n° 2012-300, 12 March 2012). Written informed consent was obtained from all consumers before their participation in the study, including a video recording consent form. The detailed aim of the study was masked in an attempt to avoid priming the consumers that incorporation of herbs and spices into low-salt legume-based recipes could give an improvement of taste and acceptance of consumers. The cover story was that the study aimed to examine the perception and appreciation of the consumer when they tasted different items of a lunch meal.

#### 2.2.2. Study Design and Test Meals

The study was conducted following a balanced randomized single blind, within-subject design from September to December 2018. Participants were asked to attend the Experimental Restaurant of Institut Paul Bocuse on five occasions, with at least a one-week interval between visits. Participants were tested on the same day of the week and at the same time of day. The first visit was a familiarization visit to the environment and experimental procedures, questionnaires, and test foods. Data recorded during this visit were not included in the results. The starter of the familiarization visit was the central point of the experimental design (0.6% *w*/*w* salt and 50% herbs and spices) to avoid potential familiarization, learning, or memory cues of the study test products and unbalancing the randomization order. 

**Figure 1:**
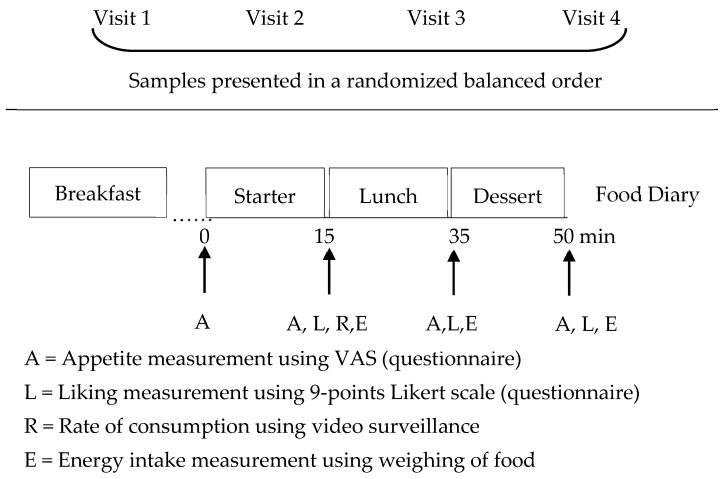
Schematic representation of the experimental procedure.

Participants arrived at 12:00 p.m. and left around 1:30 p.m. representing a typical French lunchtime. On the day before each visit, the volunteers were requested to refrain from alcohol and intensive physical activity and to consume an evening meal, similar to and at the same time before each study visit. On the day of each visit, the volunteers were requested to eat the same breakfast at the identical time as the first visit, and they were instructed to not eat any food or drink any caloric beverages in the morning. Subjects completed a questionnaire to assess compliance with these instructions and any possible factors that could influence appetite. The aim was to standardize, to the extent possible, the energy expenditure and intake, without modifying the eating habits of each consumer. A food diary was provided and filled out by consumers during the study days. On arrival at the experimental restaurant at 12:00 p.m., subjects sat down with their relatives, colleagues, or friends to simulate consumption in a real ecological context. They answered a few questions that allowed us to check compliance with the instructions of having the same dinner and breakfast, and refraining from physical activity and smoking. Appetite ratings were assessed before the mezze, which was served as a starter and at specific time intervals throughout the study, after the starter, the main dish, and dessert. The experimental day procedure is represented schematically in Figure 1.

Subjects were provided with one of the four mezzes (S, LS, SHS, and LSHS) presented as a starter dish according to a randomized weekly session sequence. The randomization method used was random permuted blocks (size of four) to allocate the order test product, which ensured test product group numbers were evenly balanced at the end of each block. Each consumer was provided a plate with 10 buckwheat salt-free crackers and a small clear glass bowl with 75 g of mezze. The typical portion size of classic humus was between 30 g and 40 g, and a double portion size was proposed in order to represent an ad libitum portion. Timing started when the subjects started eating (0 min) and they were instructed to consume until they felt comfortably satisfied within 15 min. A new plate containing 10 crackers and a bowl with 75 g of mezze was available for subjects who wanted more within the 15 min of allocated time for the starter. After 15 min, subjects were provided with one piece of commercially available beef lasagna (Metro Chefs, France) and a white bread roll (Bridor, France). One vanilla yogurt (Danone, France) and one apple (Metro, France) were also provided as dessert in the allocated time described in Figure 1. The nutritional composition of all food items is provided in Table 3. The low- and standard-salt mezzes provided 6.4% and 12% of the salt content of the entire meal, respectively. 

Appetite ratings of hunger, fullness, desire to eat and prospective consumption, overall liking, and food intake measurements were conducted immediately after consumption of each study food item provided at lunch (Figure 1). Eating behavior assessment using video surveillance was captured during starter consumption. Ad libitum room-temperature tap water was provided during the lunch, and other beverages were not allowed. Following the completion of lunch, volunteers were free to leave the experimental restaurant. They were provided with a food diary and were instructed to record all food and beverages consumed for the remainder of the study day.

#### 2.2.3. Measurements 

##### Hedonic Evaluation of the Mezzes and Study Food Items

Overall liking and liking of sensory modalities of the developed legume-based mezze and food items were assessed using a nine-point hedonic Likert scale anchored by “1 = dislike extremely” to “9 = like extremely” at the two ends. Subjects assessed overall liking followed by liking of the three sensory modalities (taste, texture, and appearance), immediately after consumption of the mezzes. Overall hedonic response to the three-course meal items was assessed right after consumption of the main dish, apple, and yogurt. 

##### Appetite Measures Using Visual Analogue Scales

The appetite profile was assessed using a 100-mm visual analogue scale (VAS) of subjective perception of hunger, fullness, desire to eat, and prospective consumption [41]. The questions were as follows: “How hungry are you?”, “How full are you?”, “How strong is your desire to eat?”, and “How much food do you think you could eat right now?”. A composite appetite score was calculated using the following formula: composite appetite (mm) = (hunger + desire to eat + (100 − fullness) + prospective consumption)/4 [42]. Lines were anchored at each end with opposing statements (e.g., 0 mm represented “I am not hungry at all”, and 100 mm represented “I am extremely hungry”). VAS ratings were recorded using pen and paper. 

##### Food and Energy Intake 

Individual study food items were weighed to the nearest 0.1 g before service to subjects and again after consumption as an indicator of how hedonic liking impacted the amount of food consumed. The total amount of food (g) and energy consumed (kJ) by the participants were determined by the weights of the individual study food items consumed during the meal. The leftovers were measured in a kitchen area out of the subjects’ sight, so that they were not aware that food was monitored. Total energy intake (kcal), and amounts of carbohydrates, lipids, and proteins were calculated based on the manufacturer’s nutritional information. Subsequent food intake and energy intake throughout the whole day (kJ) was assessed from self-reported food diaries provided to the subjects after each visit using the Nutrilog nutrition analysis software (Copyright © 2007 Nutrilog SAS, Marans, France).

##### Assessment of Eating Rate 

The mezze consumption was recorded using video devices (Sony EVI-D70) located in the ceiling of the experimental restaurant that targeted the face and plates of subjects. Only subjects fully visible in video recording during the entire mezze consumption for the four sessions were included in the data analysis (*n* = 40). The following variables were assessed from visual inspection and elaboration of the video tapes using the BORIS software (Behavioral Observation Research Interactive Software; Copyright © 2012–2019 Olivier Friard, Marco Gamba, Italy) to study the rhythm of the meal; the time of consumption of the mezze was the time from the first bite until the total consumption or until the last bite, the total number of bites during the starter, and the “eating rate” as measured by the number of bites during the consumption time of the starter, presented as bites per minutes [43,44]. Water consumption was also monitored by counting the number of sips. This variable was analyzed as a confounding factor for the number of bites but also as a dependent variable. Collectively, the temporal pattern of the mezze was entirely reconstructed and analyzed. 

##### Habitual Salt Intake 

The food frequency questionnaire (FFQ) developed by Charlton et al. (2007) [45] was used and slightly adapted to the French population to characterize subjects’ habitual salt intake. Subjects were instructed to complete this questionnaire, composed of 42 food items at the first visit, given that there is some evidence showing an association between sodium intake and salt taste sensitivity and liking for salty foods [46]. A sodium score was calculated from the sodium content per serving multiplied by the frequency factor, producing the total daily sodium intake for each subject. A cut-off score of 2400 mg of sodium per day, corresponding to a value of 48 with the simplified scoring system, was used to separate desirable and excessive sodium intake as per the authors’ instructions [45] and was used as a confounding factor in the data processing. 

### 2.3. Phase III—Follow-up Assessment Study

#### Appreciation of the Different Versions of the Selected Herb and Spice Modification Presented at the Same Session

Contrary to the phase II study, where subjects tasted the four mezzes (S, LS, SHS, and LSHS) as a starter one week apart and according to a randomized weekly session sequence, in the follow-up assessment study, the four mezzes were tasted during the same session for direct comparisons between the mezzes as an indicator of relative liking. Different subjects were recruited for phase III. In total, 121 subjects, who did not participate in phase I and phase II and without any relevant food allergies and intolerances, dislike of legumes, or health problems affecting taste or smell, were recruited using an online invitation as described earlier and enrolled in the follow up test. Written informed consent was obtained from all consumers before their participation in the study. The test was carried out in May 2019 at the experimental restaurant of the Research Center of Institut Paul Bocuse. Each participant was served with 30 g of each recipe as a starter dish at lunch (served at 12:00 p.m.). The mezzes were served individually in identical clear glass cups, numbered with a three-digit random code and presented on the same line in a balanced order (permuted blocks of four). Consumers were asked to taste the four mezzes without the need to finish any of them and to rinse their palate between samples with water and buckwheat salt-free crackers. Overall hedonic liking was scored on a nine-point Likert scale. A preference ranking test was also performed in order to ask the participants which blend was preferred, regardless of participants’ personal overall hedonic opinion.

## 3. Statistical Analysis

### 3.1. Power Analysis

The study power for the phase I and III single-session tests was calculated based on the effect sizes of previous studies examining the effect of herbs and spices on overall liking [22,24,47]. According to those data, and assuming a between-subject variance on the nine-point Likert scale to be 0.3 to detect a difference of one, 121 subjects were needed to complete the tests. Power analysis indicated that a total of 85 subjects were needed for this four-test product within subject study in phase II. The probability was 90% and with alpha 0.05, within-subject standard deviation of 1.9, and minimal detectable difference in mean overall liking of one. We obtained enrollment of 131 subjects and assumed a dropout rate of about 20%. 

### 3.2. Phase I and III

Differences in overall liking and liking of the sensory modalities scores between the blends of herb and spice modifications (phase I) and salt versions (phase III) were evaluated using a linear mixed model (ANOVA), with test product as a fixed effect and subject as a random effect. The model was adjusted for multiple comparisons using the Tukey–Kramer post hoc significance test. Statistical analysis was performed using SAS statistics software (version 9.2; SAS Institute Inc., Cary, NC, USA). 

For the preference ranking data, the Friedman test was used to evaluate which blend of herb and spice modification (phase I) and which version of the LS, S, LSHS, and SHS mezze (phase III) was ranked first. The 2-AFC tests for saltiness and spiciness were analyzed with a binomial test. Both Friedman and binomial tests were performed using XLSTAT software (Copyright © 2017. Addinsoft 2017. XLSTAT 2011: Data Analysis and Statistical Software for Microsoft Excel. Paris, France). 

### 3.3. Phase II

A repeated-measures mixed model ANOVA (PROC MIXED procedure, SAS Institute) using an autoregressive covariance structure was performed to examine the effect of the herb and spice modification on overall liking, liking of sensory modalities, appetite, energy and food intake, and eating behavior (video-captured data) for phase II. Test product (S, LS, SHS, and LSHS), visit, and time were included as fixed-effect predictors, and subject was treated as a random effect, while visit was included as a repeated effect (model 1). Further adjustment for gender, body mass index (BMI), gender × test product interaction, frequency of legume consumption, frequency of adding salt and herbs and spices (H&S) while cooking, differences in the habitual salt intake FFQ, and differences in eating restraint in the three factor scores was performed in model 2. Adjustment for individual energy needs was performed by estimating the food intake of the study food items divided by the subjects’ estimated energy requirement (EER) by the Institute of Medicine equation [48] and the activity level (hours of physical activity declared at the screening questionnaire). Further backward stepwise analysis was conducted by checking the significance of these fixed effects or their interactions and including in model 3 only the significant effects (approximate F-tests). For the appetite variables, a two-way analysis of variance (ANOVA) was also used to control that there were no differences between test products at baseline (t = 0 min).

An additional analysis for phase II and III using a general linear model was performed by dividing the test product (S, LS, SHS, and LSHS) into two factors, the level of salt (S, SHS vs. LS, LSHS), and the level of H&S (S, LS vs. SHS, LSHS) to assess the effect size of H&S and salt on liking, comparing the F-values from each factor. The interaction effect between salt and H&S factors was also tested.

The data distribution and all models’ residuals were tested for normality using standard diagnostics. Adjustment for multiple comparisons of significant effects was carried out with the Tukey–Kramer post hoc significance test. Data are presented as least square means (LSMs) and standard errors of the mean (SEM), unless otherwise indicated. A *p*-value <0.05 (two tailed) was deemed statistically significant. Statistical analysis in phase II was performed using SAS statistics software (version 9.2; SAS Institute Inc., Cary, NC, USA).

## 4. Results 

### 4.1. Phase I

#### 4.1.1. Appreciation of the Legume-Based Mezzes with Different Herb and Spice Modifications

**Figure 2:**
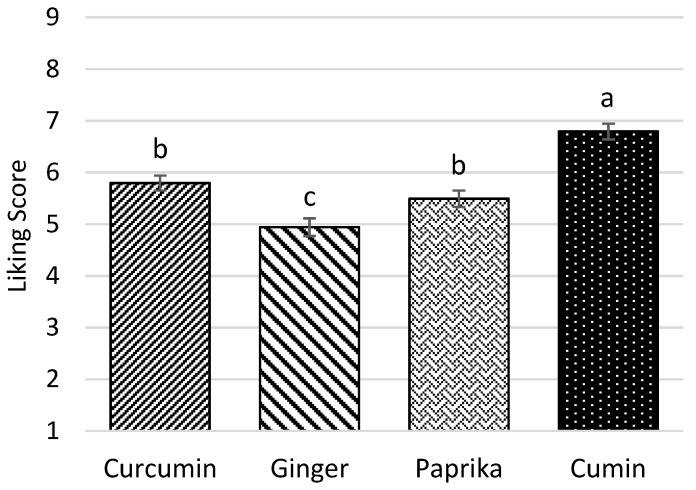
Mean overall liking scores with the standard error of the four different blends of herbs and spices based on a linear mixed model analysis (*n* = 130). ^a,b,c^ Mean values within a row with different letters were significantly different (*p* < 0.05, ANOVA followed by Tukey’s post hoc test).

In the first consumer test, the low-salt legume-based mezzes flavored with the four different blends of herbs and spices were tested to select the most liked version of the mezzes to progress in the cross-over study (Figure 2). The least and the most appreciated mezzes were the ginger (5.0 ± 0.17, *p* < 0.05) and the cumin blend modification (6.78 ± 0.15, *p* < 0.001), respectively. There was no significant difference between the curcumin and paprika blend modifications (*p* = 0.325). 

**Figure 3:**
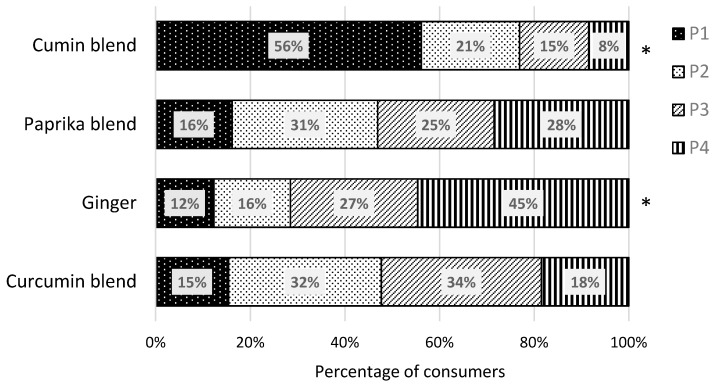
The percentage of consumers who preferred each legume-based mezze with distinct blends of herbs and spices (cumin, paprika, ginger, and curcumin blends). Significance is indicated with a star (P1, most preferred; P2, second preferred; P3, third preferred; P4, least preferred, *p* = 0.006, Q = 12.5).

Results from the forced choice preference test are summarized in Figure 3 representing the percentage of consumers (*n* = 130) who ranked the four mezzes with the different blend of herbs and spices from the most (P1) to the least preferred (P4). A Friedman test was conducted on the frequency counts for each blend, and significance was defined as *p* < 0.05. The cumin blend modification was selected by 56% (*n* = 73) of subjects as the most preferred, and the ginger blend modification was selected by 45% (*n* = 58) of subjects as the least preferred based on the frequency counts for each mezze (*p* = 0.006, Q = 12.5). 

#### 4.1.2. Directional Paired Comparisons of Perceived Salt and Spice Intensity 

The 2-AFC test showed that the low-salt cumin legume-based mezze was perceived to be significantly less salty than the standard-salt cumin legume-based mezze (*p* < 0.0001); 55 and 58 consumers out of 61 scored the standard sample as saltier, respectively, in SHS/LSHS and S/LS pairs. Moreover, a significant difference in spiciness was observed (*p* < 0.0001); 45 consumers out of 54 scored the herb and spice samples as spicier in both LS/LSHS and S/SHS pairs. These results highlight the fact that the four mezzes were easily distinguished in terms of saltiness and spiciness. 

### 4.2. Phase II

#### 4.2.1. Subject Characteristics

A total of 94 volunteers completed the cross-over study, aged 18–35 years, and 62% (*n* = 58) were women (see flow diagram in Figure S1 Supplementary Materials). In total, 72% (*n* = 68) were non-smokers, and all subjects had a BMI ranging from 17.7 to 29.9 kg/m² (9% underweight, 80% normal weight, and 11% overweight) calculated from self-reported height and weight. The mean score for Factor 1 of the Three Factor Eating Questionnaire was 5.8 ± 4.0, and the mean score for the FFQ for salt intake was 28.5 ± 14.3. Regarding appreciation of spicy cuisine, 57% declared a positive appreciation. Fifty subjects declared a high frequency (“often”, “always”) of adding herbs and spices while cooking, whereas 37 declared a low frequency (“sometimes”, “rarely”), and four declared never using herbs and spices while cooking. With regard to use of salt while cooking, 42 subjects declared a high frequency, 42 subjects declared low frequency, and four subjects declared never using salt while cooking.

#### 4.2.2. Appreciation of the Legume-Based Mezzes at Varied Salt and Herb and Spice Levels 

**Figure 4:**
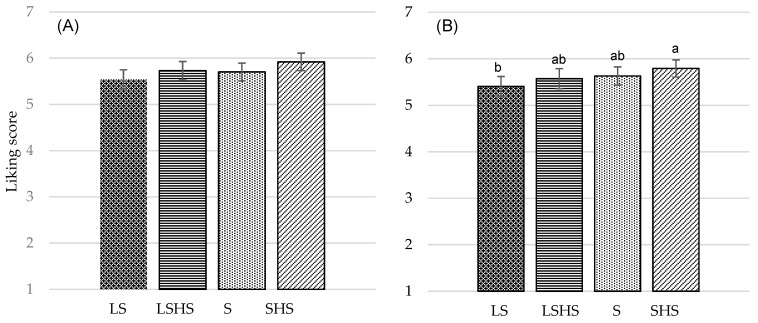
(**A**) Mean overall liking scores (mean ± standard error, SE) and (**B**) mean taste liking scores on nine-point Likert scale of the four versions of the cumin blend legume-based mezze, each being presented in different session, one week apart (absolute liking): the standard-salt legume-based mezze (S), the low-salt legume-based mezze without herbs and spices (LS), the standard-salt legume-based mezze with herbs and spices (SHS), and the low-salt legume-based mezze with herbs and spices (LSHS). ^a,b,c^ Mean values within a row with different letters were significantly different (*p* < 0.05, ANOVA followed by Tukey’s post hoc test).

Ratings for overall liking and liking of taste obtained in the cross-over study (*n* = 94) of the four different versions of the cumin blend legume-based mezze, with each version being tasted in a different session, are summarized in Figure 4A,B. Mezze received liking scores between five and six, consistent with acceptable ratings of ordinary foods, representative of what consumers would generally get in a workplace canteen. There was a test product effect for the liking of taste (*p* = 0.044) (Figure 4B). The taste score was higher for the SHS compared with the LS mezze (*p* = 0.027), whilst there were no differences between those and the S and LSHS versions (*p* > 0.05). A similar trend was observed for the effect of the mezze on overall liking, yet without reaching significance (*p* = 0.065) (Figure 4A). There were no differences in ratings for the liking of appearance and texture between the four versions of the mezze, even when the models were adjusted for several confounding factors (Table S1, Supplementary Materials). 

Results based on the level of salt and herbs and spices (two-factor analysis) showed that the effect of herbs and spices was as large as the effect of salt on overall liking by reaching borderline significance (F(1, 276) = 3.79, *p* = 0.052; F(1, 276) = 3.29, *p* = 0.071, respectively). However, the effect of salt (F(1, 276) = 4.75, *p* = 0.030) was significantly larger than the effect of herbs and spices on liking of taste (F(1, 276) = 2.33, *p* = 0.128).

#### 4.2.3. Appetite Profile

Baseline appetite ratings (hunger, fullness, desire to eat, prospective consumption, and overall appetite) (t = 0) were not significantly different between the four test products. The appetite ratings measured by VAS are shown in Figure S2 (Supplementary Materials). Over the entire lunch (t = 0 to t = 50), there was an effect of time (*p* < 0.001) and gender (*p* < 0.05), but there was no significant test product × time interaction. The means of all appetite ratings over the lunch time session, following the consumption of the four mezzes, are shown in Table 2. There were no differences between the four versions of the cumin legume-based mezze in overall appetite, hunger, desire to eat, and prospective consumption. However, fullness ratings were significantly lower in the session when the LSHS mezze was offered compared with the LS, S, and SHS mezzes (*p* = 0.009). The test product effect for fullness lost its significance when the model was further adjusted for several confounding factors related to subject characteristics (BMI, gender) and frequency of using salt, herbs, and spices (model 2). There were no changes in the test product effect in the backward stepwise analysis by excluding the variables without a significant effect (model 3). The results for the model 2 and model 3 analyses are given in Table S2 (Supplementary Materials).

#### 4.2.4. Ad Libitum Legume-Based Mezze, Salt, and Total Energy Intake

Table 2 shows the food intake of the different food test items over the lunch time session. No significant difference was observed between the mean ad libitum energy intake of the mezzes. The average energy intake of the starter (mezze + crackers) was 235 ± 88 kcal. Similarly, no significant difference was observed between the mean ad libitum energy intake of the entire lunch meal. The average energy intake of the entire meal was 838 ± 191 kcal. There were no changes in the test product effect when the model was further adjusted for possible confounding factors or after the backward stepwise analysis by eliminating the non-significant factors (Table S1, Supplementary Materials).

There were no significant differences in the energy intake based on the food diary analysis for the remainder of the day between the four study products and meals (LS: 1059 ± 70, LSHS: 1053 ± 70, S: 1045 ± 70, SHS: 1040 ± 72 kcal; *p* = 0.995). There were no significant differences between the study food items of the meal (main dish, *p* = 0.276; dessert, *p* = 0.069; total lunch energy intake, *p* = 0.220) following consumption of the four mezzes (data not shown). There were no changes in the intake outcomes when food intake of individual food items was further divided by the subjects’ total energy expenditure (EER) except for the dessert (starter/EER, *p* = 0.965; main dish/EER, *p* = 0.071; dessert/EER, *p* = 0.015; total lunch energy intake/EER, *p* = 0.208; data not shown).

Salt intake for the test meal was 5% and 7% lower after the consumption of the LS relative to S and SHS mezzes. An even bigger reduction in salt intake for the entire test meal was observed after consumption of the LSHS relative to the S and SHS mezzes (13% and 11%, respectively).

#### 4.2.5. Eating Behavior

Eating behavior was analyzed in a subset of participants (*n* = 40) during the consumption of the starter. Summarized results of the rhythm of the starter consumption are shown in Table 4. The average starter consumption was 9.17 ± 3.58 min out of 15 min, the average total number of bites during the starter was 26 ± 11 bites, and the eating rate was 3.1 ± 1.2 bites/min. Regarding water, the average number of sips during starter was 5 ± 4 sips. There was no difference in duration of consumption (from the first to the last bite) between the four test products, nor in the total number of bites and sips. However, a trend was observed for the bite rate (*p* = 0.067) with the eating rate being lower for the LS than for S and SHS mezzes. Similar results were observed for models 2 and 3 (Table S2, Supplementary Materials).

### 4.3. Phase III

#### Appreciation of the Legume-Based Mezzes at Varied Salt and Herb and Spice Levels Presented at the Same Session

**Figure 5:**
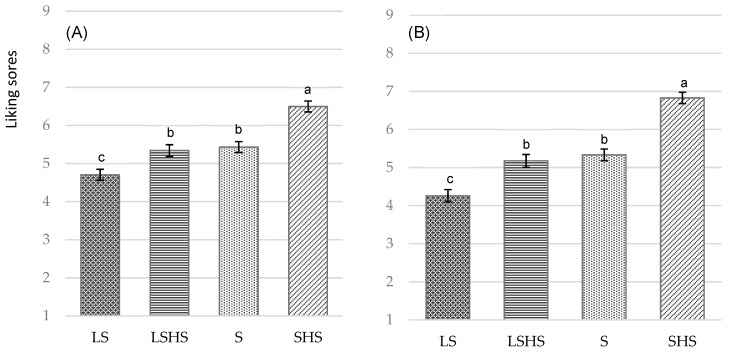
(**A**) Mean overall liking scores (mean ± SE) and (**B**) mean taste liking scores on nine-point Likert scale of the four versions of the cumin blend legume-based mezze presented at the same session (relative liking): the standard-salt legume-based mezze (S), the low-salt legume-based mezze without herbs and spices (LS), the standard-salt legume-based mezze with herbs and spices (SHS), and the low-salt legume-based mezze with herbs and spices (LSHS). ^a,b,c^ Mean values within a row with different letters were significantly different (*p* < 0.05, ANOVA followed by Tukey’s post hoc test).

Ratings for overall liking and liking of taste obtained in the confirmatory follow-up consumer test (*n* = 121) of the four different versions of the cumin blend legume-based mezze are summarized in Figure 5A,B, respectively. The least appreciated version, for overall and taste liking, was the LS (4.70 ± 0.15 and 4.26 ± 0.16, respectively), and the most appreciated was the SHS (6.50 ± 0.14 and 6.83 ± 0.15, respectively). Significant differences were observed between standard-salt and low-salt mezzes, independent of the presence of herbs and spices (*p* < 0.001). However, there was no significant difference between S and LSHS versions, either for overall liking (*p* = 0.941) (Figure 5A) or for taste appreciation (*p* = 0.830) (Figure 5B). Results from the forced choice preference test (*n* = 121) showed that the SHS version was selected by 74% (*n* = 89) of subjects as the most preferred mezze and the LS version was selected by 57% (*n* = 69) of subjects as the least preferred mezze based on the frequency counts for each mezze (data not shown).

According to the analysis on the level of salt and herbs and spices (two-factor analysis), the effect of herbs and spices was as large as the effect of salt on overall liking (F(1, 360) = 56.3, *p* < 0.001; F(1, 360) = 69.0, *p* < 0.001, respectively). However, the effect of salt (F(1, 360) = 108.8, *p* < 0.001) was larger than the effect of herbs and spices on liking of taste (F(1, 360) = 85.2, *p* < 0.001).

## 5. Discussion

To our knowledge, this is the first study to examine the effectiveness of herbs and spices to improve liking and preference for low-salt legumes conducted in an experimental restaurant setting, a controlled environment relevant to study consumers’ preferences in real-life eating situations. It is among the few studies which used two distinct test paradigms to assess the overall liking of the different versions of the legume-based mezze modified in terms of saltiness and spiciness at different sessions (absolute liking) and during the same session (relative liking). The findings of the present study showed that the standard-salt and low-salt with herbs and spices mezzes were equally liked at both test paradigms. Although there were some differences in the relative overall liking between seasoned and unseasoned mezzes, there were no differences for the absolute overall liking in the cross-over design. This was also reflected in lack of a significant difference in appetite ratings and consumption of mezze or of the entire meal between the four sessions.

To date, a few studies examined the influence of seasoning to increase the liking and consumption of foods with lower palatability such as vegetables, mainly in children or adolescents, with contrasting results [26,28,29,47]. Fritts et al. (2018) [47] compared the liking scores of eight seasoned and the equivalent unseasoned (oil and salt) vegetables among 110 high-school students. The seasoning significantly improved the liking and the preference for several vegetables relative to the plain varieties when served at the school lunch. However, in a follow-up short-term study, they failed to show an effect of herbs and spices on increasing the students’ vegetable consumption, although, as the authors suggested, with short repeated exposure, the willingness to consume these flavors might have been increased [26]. Manero et al. [28] showed no difference in liking scores between seasoned and steamed versions of vegetables (carrot, broccoli, and green bean) when served as a side dish in a public café on a university campus. In most of the studies, vegetable consumption was measured in an aggregated form; thus, individual intakes could not be tracked.

There is only one published study that looked at the influence of the variety of seasoning in legumes and particularly bean consumption in a population-based case control study of Costa Rican adults [49]. Findings revealed that increasing the variety of seasoning was associated with bean intake and, thus, a potentially appropriate strategy to improve intake of legumes. Legume incorporation in peoples’ eating plans includes several barriers mainly due to personal taste preferences and lack of recognition and unfamiliarity by consumers [37,50,51]. This led to the creation of the International Year of Pulses campaign in 2016 by the Food and Agriculture Organization of the United Nations [52]. Legume offerings are minimal in non-ethnic restaurants and account for only 9% of legume intake [53]. Developing recipes as a spread and offering it as a mezze was a way to increase the feasibility and likelihood that legumes could be broadly adopted to similar eating environments including school and work canteens.

A few studies explored the potential role of culinary herbs and spices and their role as flavor enhancers in low-salt foods [22,23,54,55]. Contrary to our findings on absolute liking in phase II, Bouhlal et al. [23] showed a strong preference toward the salted versions relative to unsalted versions of green beans and pasta, with a clear impact of salt content on the intake of those foods. However, as the authors stated, the impact was different for the type of foods with salt reduction, decreasing the intake of green beans by 21%, and addition of salt, increasing intake of pasta by 24%. This perception of saltiness and preference for a salty taste might be food- or product-specific as previously shown [46,56,57]. However, the consumption of pasta did not differ between the 0% and 0.6% added salt variant in children despite the differences in hedonic evaluation between those products, supporting previous findings in adults showing an overestimate of the ideal salt content in foods based only on sensory evaluation [55]. In our cross-over study, there were no differences in overall appreciation between the low-salt and the standard-salt mezzes, which was accompanied by no differences in food intake. However, in the follow-up assessment, when all the mezzes were presented at the same session, as an indicator of relative liking, both overall appreciation and liking of taste were significantly higher for the standard-salt mezze with added herbs and spices and lower for the low-salt mezze, and no significant difference was found between the low-salt with herbs and spices and the standard-salt mezzes. Those results are partially in agreement with Gwahi et al. [22], who showed the liking of taste to be significantly lower in the low-salt soup. However, contrary to our findings, which showed that liking was enhanced by incorporating herbs and spices in the low-salt mezze, Gwahi et al. [22] reported no difference between the low-salt soup and the low-salt soup with added herbs and spices. This could be due to differences in food type and food-specific salty preference as mentioned earlier, or differences in herb and spice modification given that certain herbs and spices contain volatile compounds that could enhance cross-modal saltiness perception [58].

Flavor is described as the combination of taste (gustation), smell (olfaction), and chemical burn (trigeminal sensation) [21]. Using the blend of herbs and spices to enhance the flavor of the mezze stimulated different aspects of the sensory system involved in flavor perception (i.e., salt for gustation, cumin for olfaction, and garlic for the trigeminal sensory system). That could possibly explain why the effect of salt was larger on the liking of taste and why the effect of herbs and spices (1.6 g) was as large as the effect of salt on overall absolute and relative liking [59,60]. These results suggest culinary actions to reduce salt content that are acceptable from a catering operator’s point of view, as changing the recipe is possible without lowering consumers’ satisfaction in real-life eating situations. A 50% salt reduction in the mezze recipe with herbs and spices resulted in a 13% reduction in salt for the total test meal, implying that the use of herbs and spices at major eating events, such as lunch and dinner, is a promising approach to reach the WHO target of a relative 30% reduction in salt intake by 2025 [11].

Among the strengths of this study was the use of two distinct test paradigms: one for absolute liking and one for relative liking in phase II and phase III, respectively. Different findings were produced in those studies that could be explained by the fact that, in the cross-over study, the one-week washout period might have been effective in blinding participants to the variation of salt and herbs and spices content compared with the side-by-side test of mezzes. In addition, individual differences in experiences and expectations with the test foods with regard to the familiarity of herb and spice blend modification and how the test foods are commonly prepared and consumed could also explain the differences in responses [47,61].

Reducing salt and adding herbs and spices in a typical consumed food, such as a hummus-type mezze in our study, generates a novel stimulus [62], which increases the unfamiliarity and sensory complexity and could reduce liking relative to the standard version, but for which repeated exposure might increase both familiarity and liking of the reduced-salt version [22]. Thus, the repeated exposure of the mezzes with similar attributes, except for the salt and herb and spice content, in the cross-over study could have induced a certain degree of familiarity compared with the single exposure. Indeed, introduction of all the test mezzes at the same session could have augmented the perception of the novel modifications. There is some evidence that intake of food can be decreased by repeated exposure to the same food for several days even if the food was initially appreciated [63]. However, there were no differences in food intake in the current study between the sessions, which could have been due to the balanced randomization and the short exposure course followed in this study.

There were no differences in overall appetite and most of the specific appetite ratings among the four legume-based mezzes. Some limited evidence exists regarding the potential effects of culinary herbs and spices on appetite and food intake regulation, via modulating appetite-related gut hormones and thermogenic effects through sensory stimulation or through the role of their bioactive and flavor compounds on digestive processes [30,32]. However, the form (e.g., oral or capsule administration) and the amount of herbs and spices are important factors considering their effect on appetite [32], with most of the studies providing a dose of herbs and spices much higher (3–20 g) than the amount of herbs and spices in the legume-based mezzes (1.6 g) [30,64,65]. Furthermore, the mezzes were similar in macronutrients, energy, energy density, texture, and legume content, factors that could influence appetite and food intake regulation [66,67] and could potentially surpass any impact of spices on appetite through activation of gastrointestinal and chemosensory signals.

A strength of this study is that it was conducted in an ecological environment representing a usual out-of-home eating context. Yet, the lack of controlled experimental conditions for the sensory evaluation and presence of other people in the table should be considered in the interpretation of the results. Another limitation is that the present findings cannot be generalized to other populations and cultures with exposure and preferences to different spices and frequency of legume consumption. Furthermore, this study included measures of reported liking, preferences, and intake of the legume-based mezze served as a starter, a part of a meal typically served in French restaurants, for better understanding of individual variations in eating behavior. However, expectation of the following parts of the meal could have influenced ad libitum food intake of the legume-based mezze with unknown results if the legume test meal would have been served in a different form and as a single main meal. Finally, salt consumption, saltiness sensitivity, and liking of salty foods are linked and impact one another [46], and, although dietary salt consumption was considered in the analysis, assessment of dietary sodium was based on FFQs, which is prone to underestimation relative to the most reliable 24-h urinary sodium analysis.

## 6. Conclusions

Overall appreciation and taste scores were similar between the low-salt with added herbs and spices and the standard-salt legume-based mezze, without differences in food consumption. These findings suggest that incorporating herbs and spices into reduced-salt food items is a feasible strategy for achieving a 50% reduction in salt content without sacrificing the hedonic liking of low-salt legume-based mezzes in a real restaurant setting. The outcome is relevant to public health efforts to reduce salt in foods eaten out of home or commercially prepared products, and it illustrates a strategy incorporating legumes in novel, convenient, and healthy food products. Further studies are needed to extend these findings to different types of legumes, food forms, and compositions, and to longer-term exposure. A greater understanding of the inter-individual differences and consumer segmentation in terms of eating behavior variables that influence appreciation of legumes and herbs and spices seems prudent.

## Figures and Tables

**Table 1:** The amount of ingredients used for developing the legume-based mezze.

Ingredients	Quantities
Cooked chickpeas (Sabarot, France)	371 g
Cooked red lentils (Sabarot, France)	159 g
Olive oil (18:1, Alexis Munoz, France)	185 g
Tahini (spread made from ground sesame seeds, Al Wadi Al Akhdar, France)	37 g
Lemon juice (pressed from fresh lemon, Bail distribution, France)	45 g
Water	203 g

**Table 2:** Inclusion and exclusion criteria of the study participants.

Inclusion Criteria	
Gender	50% men/50% women
Age	18–35 years inclusive
Body mass index, BMI (kg/m²) ^1^	18.5–30 kg/m² inclusive
**Exclusion Criteria**	
Participation in phase I tests	
Special diet (on weight loss diet, vegetarian, or any dietary restriction)	
Food allergies or intolerances to study products	
Pregnancy or breastfeeding	
Any health problems that could affect taste, smell, or appetite	
Cognitively restrained eaters ^2^	
Athletes in training (>10 h of sport/week)	

^1^ BMI was self-reported and is defined as the body weight divided by the square of the body height. ^2^ TFEQ, the three-factor eating questionnaire, with cognitive restraint eating (score range 0–21); cut-off point was 11, with scores above 11 showing cognitive restraint [40].

**Table 3:** Energy content and macronutrient composition of the food items and entire meal provided during the study session.

Nutritional Values per 100 g and Serving Portion	Crackers		Mezze *		Lasagna		Bread		Yogurt		Apple		Meal
	100	28	100	75	100	400	100	40	100	125	100	85	753
Energy (kcal)	388	111	217	163	132	528	253	101	93	116	54	46	1065
Fat (g)	2.7	0.8	18.5	13.9	5.5	22	0.8	0.3	3.1	3.9	0.25	0.2	41.1
Saturated fat (g)	0.6	0.2	2.5	1.9	2.4	9.6	0.2	0.08	2	2.5	0.05	0.04	14.3
Carbohydrates (g)	75.2	21.4	8.9	6.7	13	52	52	20.8	12.6	15.7	11.6	9.9	126.5
Sugars (g)	1.7	0.5	0.6	0.5	2.3	9.2	0.8	0.3	12.6	15.7	9.3	7.9	34.1
Fibers (g)	4.5	1.3	2	1.5	0	0	2.9	1.2	0	0	1.4	1.2	5.2
Proteins (g)	13.4	3.8	4.5	3.4	6.8	27.2	7.9	3.2	3.6	4.5	0.25	0.21	42.3
Salt (g) ^1^	0.01	0	0.01	0	0.9	3.6	1.6	0.6	0.13	0.2	0	0	4.4

Energy and macronutrient content was calculated using the table of food nutrition composition (Ciqual) from ANSES (Agence nationale de sécurité sanitaire de l’alimentation, de l’environnement et du travail) and/or information on food labels. * Nutritional values of the study mezze were calculated using the Nutrilog software. ^1^ Salt content of the entire meal with the low-salt mezze.

**Table 4:** Mean subjective appetite responses using visual analogue scale (VAS), intake of the test food items in grams, and eating behavior components over the study day after the consumption of mezzes as a starter (mean values with standard errors).

	Test Products								
	LS		LSHS		S		SHS		
	Mean	SE	Mean	SE	Mean	SE	Mean	SE	*p*-Value
***Appetite profile*** ^1,2^									
Hunger (mm)	28.2	1.2	29.4	1.2	27.9	1.2	30.4	1.2	0.209
Fullness (mm)	67.7 ^a^	1.5	63.1 ^b^	1.5	68.8 ^a^	1.5	66.0 ^a,b^	1.5	0.009
Desire to eat (mm)	27.5	1.3	28.0	1.3	27.3	1.3	30.2	1.3	0.143
Prospective intake (mm)	26.6	1.3	27.5	1.3	27.2	1.3	28.7	1.3	0.417
Overall appetite (mm)	28.6	1.1	30.4	1.1	28.4	1.1	30.8	1.1	0.106
***Intake*** ^1^									
Legume mezzes (g)	63.4	2.9	64.5	2.9	64.4	2.9	63.2	2.9	0.864
Salt-free crackers (g)	22.8	1.0	21.8	1.0	20.8	1.0	22.6	1.0	0.084
Starter’s energy (kcal)	237	9	236	9	231	9	235	9	0.870
***Eating behaviour*** ^1^									
Eating rate (bites/min)	2.8	0.2	3.0	0.2	3.3	0.2	3.2	0.2	0.067
Number of bites	25.6	1.8	26.1	1.8	26.1	1.8	27.2	1.8	0.657
Number of sips	5.2	0.6	4.6	0.6	5.5	0.6	5.6	1.0	0.329
Consumption time (min)	9.1	0.6	8.5	0.6	8.4	0.6	9.0	0.6	0.576

^1^ The model was adjusted for visit, test product, and time for all outcomes; ^2^ for the appetite profile variables, the model was further adjusted for the baseline values. S, standard-salt legume-based mezze; LS, low-salt legume-based mezze without herbs and spices; SHS, standard-salt legume-based mezze with herbs and spices; LSHS, low-salt legume-based mezze with herbs and spices. ^a,b,c^ Mean values within a row with different superscript letters were significantly different (*p* < 0.05, ANCOVA followed by Tukey’s post hoc test). SE, standard error.

## Supplementary Materials

The following are available online at https://www.mdpi.com/2072-6643/11/12/2901/s1, Table S1: Data on overall liking and liking of taste, appearance, and texture scores in the cross-over study; Table S2: Data on further adjusted models for appetite profile, intake, and eating behavior variables following consumption of the four mezze meals for the cross-over study; Figure S1: Participants’ flow diagram; Figure S2: Subjective appetite profile VAS ratings.
