# Supplementary material for: The Impact of Herbs and Spices on Increasing the Appreciation and Intake of Low-Salt Legume-Based Meals
Source: Nutrients. 2019 Dec 1;11(12):2901. doi: 10.3390/nu11122901 (PMC6950109; doi:10.3390/nu11122901)
Supplement: Supplementary file 1 [file nutrients-11-02901-s001.pdf]

Online Supplementary material

Table S1. Mean overall liking and liking of taste, appearance, and texture scores (mean ± SE) on nine-point Likert scale of the four versions of the cumin blend legume-based mezze, each being presented in different sessions, one week apart.

	Test product								
	LS		LSHS		S		SHS		
	Mean	SE	Mean	SE	Mean	SE	Mean	SE	P for trend
Overall Liking									
Model 1	5.54	0.20	5.73	0.20	5.72	0.20	5.92	0.20	0.065
Model 2	5.49	0.65	5.66	0.65	5.58	0.65	5.78	0.65	0.254
Liking of taste									
Model 1	5.39^{b}	0.20	5.56^{a,b}	0.20	5.63^{a,b}	0.20	5.81^{a}	0.20	0.044
Model 2	5.37	0.66	5.58	0.66	5.51	0.66	5.71	0.66	0.149
Liking of texture									
Model 1	5.61	0.20	5.72	0.20	5.75	0.20	5.64	0.20	0.809
Model 2	5.56	0.62	5.70	0.62	5.54	0.62	5.46	0.62	0.567
Liking of appearance									
Model 1	5.69	0.19	5.47	0.19	5.61	0.19	5.67	0.19	0.371
Model 2	5.57	0.60	5.33	0.60	5.37	0.60	5.44	0.60	0.388

Model 1 was adjusted for treatment, visit, and time.

Model 2 was further adjusted for gender, BMI, gender × treatment interaction, frequency of legume consumption, frequency of adding salt while cooking, frequency of adding herbs and spices (H&S) while cooking, score for the Salt Intake questionnaire, and score for the TFEQ Factor 1.

S, standard-salt legume-based mezze; *LS*, low-salt legume-based mezze without herbs and spices; *SHS*, standard-salt legume-based mezze with herbs and spices; *LSHS*, low-salt legume-based mezze with herbs and spices.

^{a,b,c} Mean values within a row with different superscript letters were significantly different ($p < 0.05$, ANCOVA followed by Tukey’s post hoc test)

Table S2. Mean subjective appetite responses using VAS, intake of the test food items in grams, and eating behavior components over the study day after the consumption of mezzes as a starter (mean values with standard errors).

	Treatment								
	LS		LSHS		S		SHS		
	Mean	SE	Mean	SE	Mean	SE	Mean	SE	P value
Appetite profile									
Hunger (mm)									
Model 2	29.8	3.4	31.1	3.4	29.4	3.4	31.4	3.4	0.841
Model 3	29.2	1.7	30.5	1.7	28.9	1.7	31.0	1.7	0.771
Fullness (mm)									
Model 2	67.0	4.1	62.6	4.1	68.4	4.1	65.8	4.1	0.236
Model 3	66.8	2.0	62.1	2.0	67.8	2.0	65.4	2.0	0.170
Desire to eat (mm)									
Model 2	29.2	3.3	29.9	3.3	28.7	3.3	31.4	3.3	0.746
Model 3	28.5	1.7	29.0	1.7	28.2	1.7	30.1	1.7	0.736
Prospective intake (mm)									
Model 2	28.2	3.2	29.2	3.2	29.1	3.2	30.5	3.2	0.835
Model 3	27.7	1.6	28.4	1.6	28.5	1.6	29.7	1.6	0.843
Overall Appetite									
Model 2	29.9	3.22	31.8	3.2	29.7	3.2	31.7	3.2	0.732
Model 3	29.6	1.6	31.4	1.6	29.4	1.6	31.4	1.6	0.707
Intake									
Legume mezzes (g)									
Model 2	70.3	8.4	69.9	8.3	69.6	8.4	68.8	8.4	0.880
Model 3	65.7	2.7	66.1	2.7	65.3	2.7	63.7	2.7	0.642
Salt free crackers (g)									
Model 2	22.8[a]	2.6	21.6[a,b]	3.0	20.3[b]	3.0	22.3[a]	3.0	0.026
Model 3	23.4	1.0	22.4	1.0	21.4	1.0	23.1	1.0	0.084
Starter's energy (kcal)									
Model 2	253	26	247	26	241	26	247	26	0.386
Model 3	245	9	242	9	237	9	239	9	0.645
Eating behavior									
Eating rate (bites/min)									
Model 2	2.8	0.3	2.7	0.3	3.1	0.3	3.0	0.3	0.109
Model 3	2.9	0.2	2.9	0.2	3.3	0.2	3.2	0.2	0.114
Number of bites									

Model 2	28.6	3.4	27.2	3.4	28.1	3.4	28.5	3.4	0.667
Model 3	26.4	1.6	26.2	1.7	26.6	1.6	27.4	1.6	0.794
Number of sips									
Model 2	4.6	1.2	3.9	1.2	4.9	1.2	4.8	1.2	0.350
Model 3	5.3	0.6	4.7	0.6	5.6	0.6	5.7	0.6	0.335
Consumption time (min)									
Model 2	11.3	1.1	10.5	1.1	10.5	1.1	11.0	1.1	0.440
Model 3	9.1	0.6	8.6	0.6	8.5	0.6	9.1	0.6	0.621

Model 2: adjusted for visit, test product, and time for all outcomes and additionally for the baseline values for the appetite profile variables, gender, overall liking, taste liking, BMI, gender × test product interaction, frequency of legume consumption, frequency of adding salt while cooking, frequency of adding H&S while cooking, score for the Salt Intake questionnaire, and score for the TFEQ Factor 1.

Model 3 was the same as model 2, excluding the variables without a significant effect.

S, standard-salt legume-based mezze; *LS*, low-salt legume-based mezze without herbs and spices; *SHS*, standard-salt legume-based mezze with herbs and spices; *LSHS*, low-salt legume-based mezze with herbs and spices.

[a,b,c] Mean values within a row with different superscript letters were significantly different ($p < 0.05$, ANCOVA followed by Tukey's post hoc test).

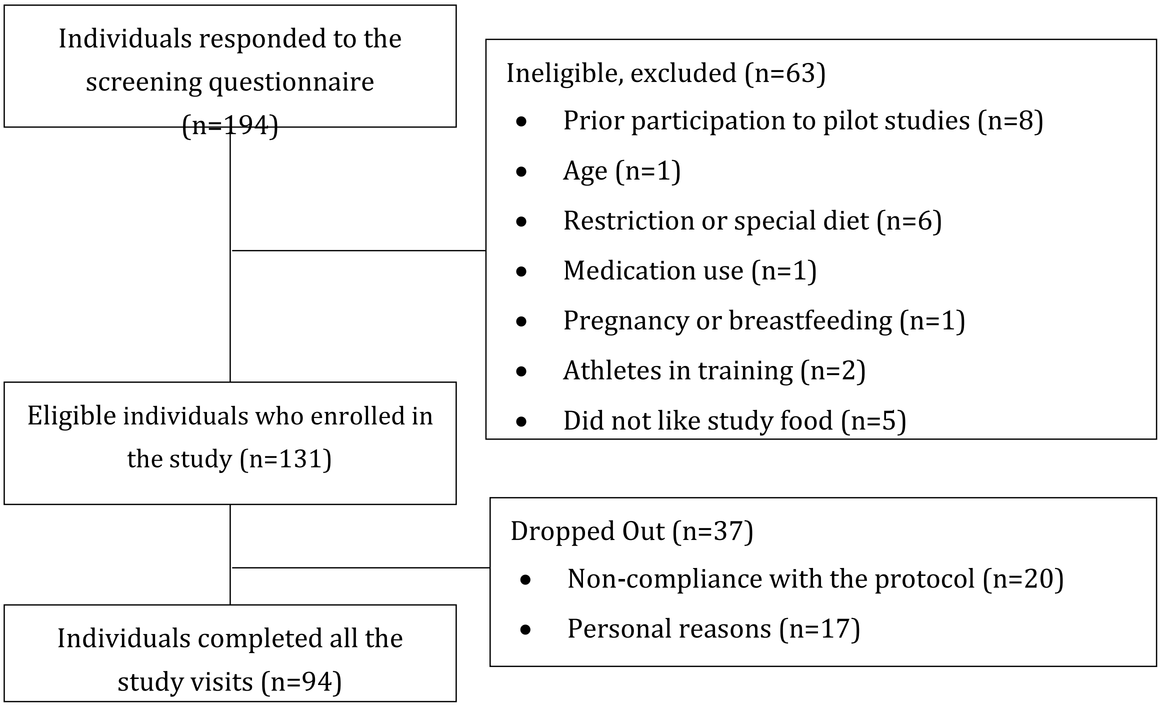

Figure S1. Participants' flow diagram of the cross-over study in phase II.

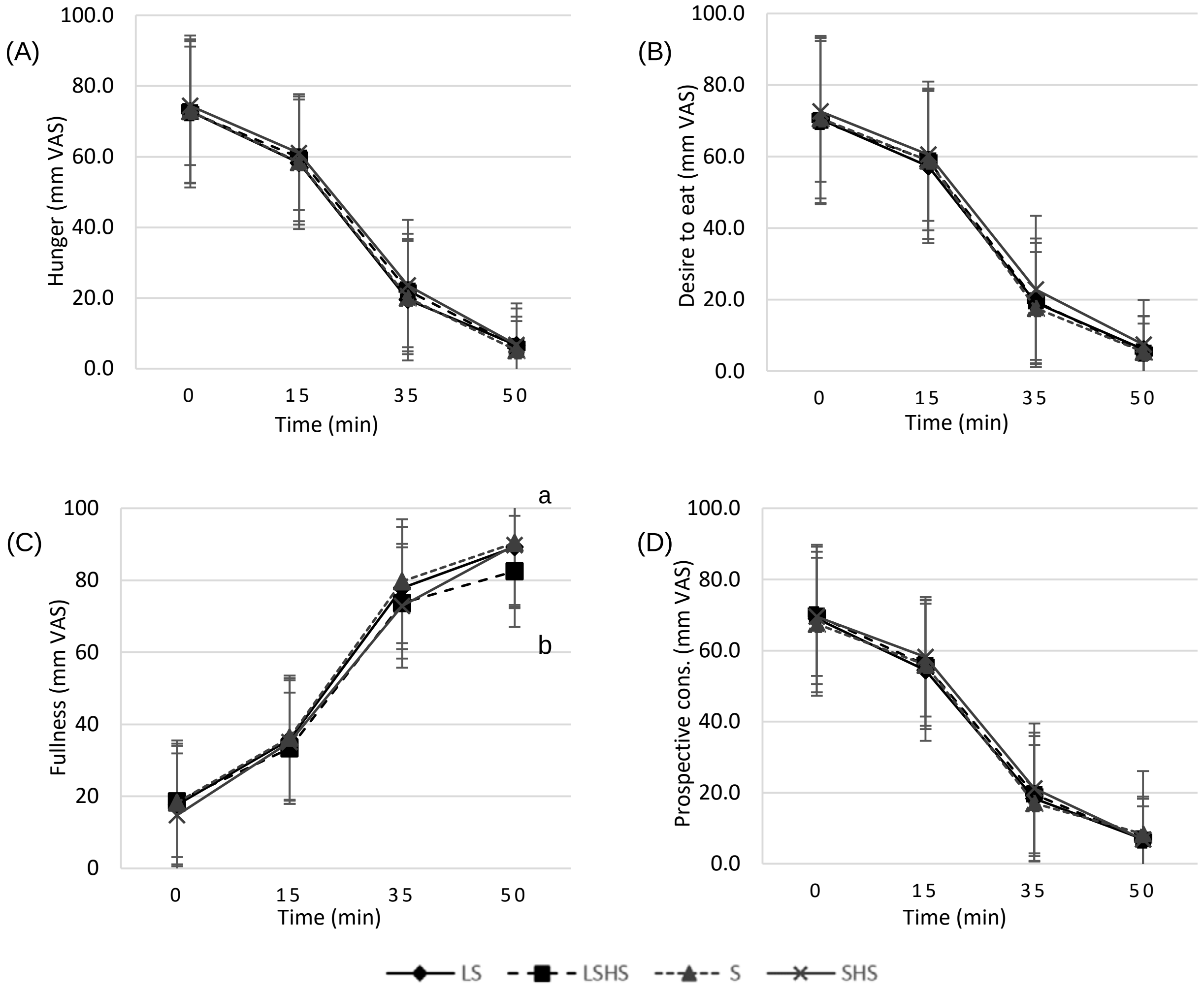

Figure S2. Subjective visual analogue scale (VAS) ratings in mm for (A) hunger, (B) desire to eat, (C) fullness, and (D) prospective food consumption throughout the study following the intake of the four versions of the cumin blend legume-based mezze, each being presented in different sessions, one week apart. S, standard-salt legume-based mezze; LS, low-salt legume-based mezze without herbs and spices; SHS, standard-salt legume-based mezze with herbs and spices; LSHS, low-salt legume-based mezze with herbs and spices. Mean values (with their standard deviation represented by vertical bars) with different superscript letters at each time point were significantly different ($p < 0.05$, ANCOVA followed by Tukey's post hoc test).
